# Scaling Wireless Continuous Vital Sign Monitoring Across an 8-Hospital Health System: Digital Health Implementation Report

**DOI:** 10.2196/78216

**Published:** 2026-01-26

**Authors:** Ngoc-Anh Nguyen, Grace Lee, Brendan Holderread, Terrie Holman, Sarah Pletcher, Roberta Schwartz

**Affiliations:** 1 Center for Connected Care, Innovation & Implementation - Research Houston Methodist Houston, TX United States; 2 Houston Methodist Research Institute Houston Methodist Houston, TX United States; 3 Department of Medicine Houston Methodist Houston, TX United States; 4 Department of Surgery Houston Methodist Houston, TX United States

**Keywords:** continuous vital sign monitoring, remote patient monitoring, wearable devices, hospital workflow redesign, patient safety, digital health implementation, implementation science

## Abstract

**Background:**

Frequent vital sign (VS) monitoring is central to inpatient safety but is traditionally performed manually every 4 hours, a century-old practice that can miss early clinical deterioration, disrupt patient sleep, and impose a heavy documentation burden on nursing staff. Continuous VS monitoring (CVSM) using wearable remote patient monitoring devices enables near real-time, high-frequency VS measurement while reducing manual workload and preserving patient rest.

**Objective:**

This implementation report describes the large-scale implementation of CVSM across an 8-hospital health system. The initiative aimed to (1) enhance earlier detection of patient health deterioration through continuous, algorithm-driven monitoring; (2) improve nursing workflow efficiency by reducing reliance on manual VS checks; and (3) minimize nighttime disruptions to support patient rest and recovery.

**Methods:**

The program was designed for system-wide scalability and executed from 2022 to 2024 using a 4-phase framework: strategic program design, program planning, go-live preparation, and implementation and optimization. A Food and Drug Administration–cleared wearable device (BioButton) continuously measured heart rate, respiratory rate, and skin temperature, with data integrated into Epic and monitored 24×7 through a centralized virtual operations center. Rollout followed a staggered playbook across approximately 2700 adult non–intensive care unit beds and was supported by leadership engagement, supply chain readiness, staff training, and phased superuser-led adoption.

**Implementation (Results):**

All 8 hospitals achieved full deployment between April 2023 and February 2024, with more than 95% device use rates and 100% nursing staff training completion. A standardized escalation workflow filtered approximately 50% of the alerts at the virtual operations center review stage, substantially reducing frontline alert burden. Operational refinements included revised heart rate and respiratory rate alert thresholds and the removal of temperature as a single alert trigger. Several units extended overnight manual VS intervals from every 4 hours to every 6 to 8 hours, with staff estimating approximately 4 hours saved per nursing shift. Patient care assistants redirected time toward patient mobility and personal care needs, while staff reported growing confidence in device performance over time.

**Conclusions:**

This initiative represents the first system-wide deployment of CVSM across a diverse, multihospital health system. Success was enabled by early strategic alignment, phased rollout, robust IT and monitoring infrastructure, and iterative optimization. The program demonstrates the feasibility of embedding CVSM into routine inpatient care to improve efficiency and patient experience. Transferable strategies, including phased rollouts, centralized monitoring, and structured change management, may inform other health systems pursuing digital VS redesign. Future work should rigorously evaluate impacts on patient outcomes, cost-effectiveness, and applicability to postacute and ambulatory care settings.

## Introduction

### Context

This implementation occurred within Houston Methodist (HM), an 8-hospital health system in Texas encompassing the HM Hospital (a quaternary care academic flagship), 6 community hospitals, and 1 long-term acute care (LTAC) facility serving the greater Houston area, together serving a diverse adult population of more than 140,000 annual admissions and 2 million outpatient visits [1].

During the COVID-19 pandemic, HM established a centralized virtual operations center (VOC) and virtual intensive care unit (VICU) providing 24×7 monitoring for approximately 370 intensive care unit (ICU) beds. This infrastructure created favorable conditions for remote patient monitoring (RPM) and ultimately informed the development of the system-wide continuous vital sign (VS) monitoring (CVSM) program. This CVSM program differs from traditional RPM in its continuous, hospital-based, and fully integrated design, with data integrated into the electronic medical record and supported by 24×7 centralized oversight rather than periodic outpatient review.

In 2022, the health system launched a redesign of inpatient VS practices, with this report detailing the program’s scale-up and deployment, which were designed from the outset for system-wide implementation.

### Problem Statement

Frequent monitoring of VSs—heart rate (HR), respiratory rate (RR), temperature, blood pressure, and oxygen saturation (SpO_2_)—is a cornerstone of inpatient safety [2]. On general hospital floors, VSs are routinely collected manually every 4 hours, reflecting a century-old practice that does not account for patient acuity [3,4]. This approach risks missed deterioration between checks, places substantial time demands on nursing staff, and disrupts patients' sleep, with physiological consequences [5-14]. Deteriorating VSs are well-established predictors of ICU transfer and in-hospital mortality, and CVSM, enabled by wearable RPM devices, now allows for unobtrusive, high-frequency VS measurement [7,11,12]. This work directly addresses health system challenges of limited access to timely clinical data and variable adherence to evidence-based monitoring practices, both of which undermine patient safety and workflow efficiency. However, evidence on broad, system-wide implementation across diverse hospital settings remains limited.

### Similar Interventions

Previous CVSM implementations have typically been limited to ICUs, single hospitals, or narrow patient groups, demonstrating feasibility but limiting generalizability [15-17]. Our initiative differs by extending CVSM across all adult non-ICU inpatients in an 8-hospital system, integrated with Epic and supported by centralized monitoring. This report describes the implementation and scale-up process.

This report follows JMIR’s implementation reporting guidelines for digital health implementation [18] to ensure transparency in describing the implementation context and processes.

## Methods

### Aim and Objective

The aim of this initiative was to redesign inpatient VS measurement practices to enhance patient safety, patient experience, and staff efficiency through the implementation of CVSM. Specific objectives were to enable early detection of deterioration, reduce nighttime disruptions to promote patient rest and recovery, and improve workflow by decreasing reliance on manual VS checks. Implementation outcomes included system-wide rollout to all eligible beds, 100% nursing staff training completion, optimal device use, and reductions in manual VS collection, with qualitative observations of staff and patient acceptance. Operational metrics (scale-up timeline, device use, alert filtering, and training completion) were directly measured, whereas workflow impacts and staff acceptance were reflected in qualitative, staff-reported estimates.

### Implementation Strategy

**Figure 1:**
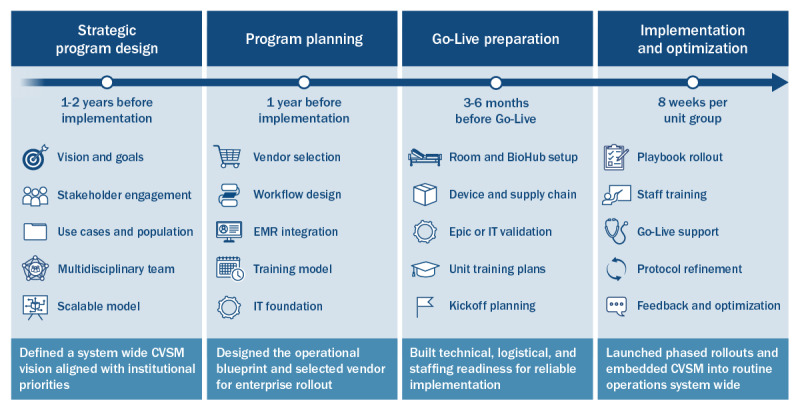
Four-phase implementation framework and observed timeline for scaling continuous vital sign monitoring across (CVSM) Houston Methodist’s 8-hospital health system. EMR: electronic medical record.

The implementation strategy was designed for system-wide scalability and organized into 4 phases, with the observed timeline shown in Figure 1.

#### Phase 1: Strategic Program Design (1-2 Years Before Implementation)

The health system defined an enterprise-wide vision for CVSM as part of a broader effort to modernize VS monitoring. Strategic goals emphasized embedding continuous monitoring into routine inpatient care, strengthening patient safety through earlier detection of deterioration, and redesigning workflows to mitigate the impact of nursing staff shortages.

A multidisciplinary team of key stakeholders, including clinical, operational, IT, supply chain, and innovation leaders, was engaged early and consistently to ensure clinical and organizational alignment. The team identified use cases and target populations, prioritizing continuous monitoring of all hospitalized, non-ICU patients to address the safety gap created by every-4-hour manual VS checks. Patient eligibility was intentionally broad; CVSM was applied to general adult medical-surgical inpatients, excluding only ICU patients, with no additional eligibility restrictions.

Implementation sites included the academic flagship, 6 community hospitals, and 1 LTAC facility, with monitoring supported by bedside nurses, unit superusers, and the centralized VOC. The multidisciplinary team guided planning and iteratively refined a scalable model for system-wide deployment, supported by superusers at the unit level and centralized monitoring through the VOC.

#### Phase 2: Program Planning (1Year Before Implementation)

After defining the overall CVSM strategy, the health system conducted market research to evaluate vendor partners and established planning priorities in workflow redesign, Epic integration, staff training, and IT infrastructure, with a standardized playbook to guide hospital rollouts. Vendor selection considered Food and Drug Administration (FDA) clearance, performance, usability, multiparameter monitoring, battery life, patient comfort, Epic integration, and scalability.

A wearable patch device (BioButton; BioIntelliSense) was selected as the FDA-cleared option that best met these criteria. The adhesive patch is worn on the chest with a battery life lasting 7 to 16 days. It continuously collects HR, RR, and skin temperature (additional parameters are listed in Multimedia Appendix 1) [6,17]. Device selection prioritized accurate RR measurement, given its established predictive value for deterioration [14,19]. Although a BioButton device does not measure SpO_2_ or blood pressure, these parameters were acceptable through existing bedside monitoring. Continuous SpO_2_ monitoring typically requires a tethered probe that disrupts rest and increases alarm burden [20], whereas the BioButton device allows unobtrusive, trended monitoring aligned with program goals to reduce patient disruptions.

From the outset, the program was designed for system-wide scale, leveraging existing institutional resources and establishing a centralized monitoring team at the main academic hospital. Pilot sites included 2 internal medicine units, 1 neurosurgery unit, and 1 neurology unit selected for innovation experience and staff receptiveness. Program management included site-level and system-level leads, who shared responsibilities with the vendor for workflow design, data integration, training, and support **(**roles are detailed in Multimedia Appendix 2).

Participating entities included HM, a quaternary academic health system whose mission emphasizes patient-centered, digitally enabled care, which led workflows, monitoring staff, and supply chain integration, and BioIntelliSense, which provided devices, hubs, dashboard tools, and technical support. The VOC delivered continuous monitoring, with bedside nurses serving as superusers. Intellectual property for the BioButton device remains with BioIntelliSense, while workflows, data integration, and the operational model belong to HM.

The business case projected nursing and patient care assistant (PCA) time savings from extended overnight VS intervals, supporting adoption and sustainability. Internal funding was secured as part of routine service redesign, with no direct costs to patients. Budget categories included devices, hubs, Epic integration, staff training, supply chain processes, and change management. BioButton devices were supplied through a per-bed subscription model, and approximately 2711 BioHub Wi-Fi gateways were installed across all patient rooms. The largest investments were technology procurement and integration, with ongoing costs for training and supply chain support.

#### Phase 3: Go-Live Preparation (3-6 Months Before Going Live)

**Figure 2:**
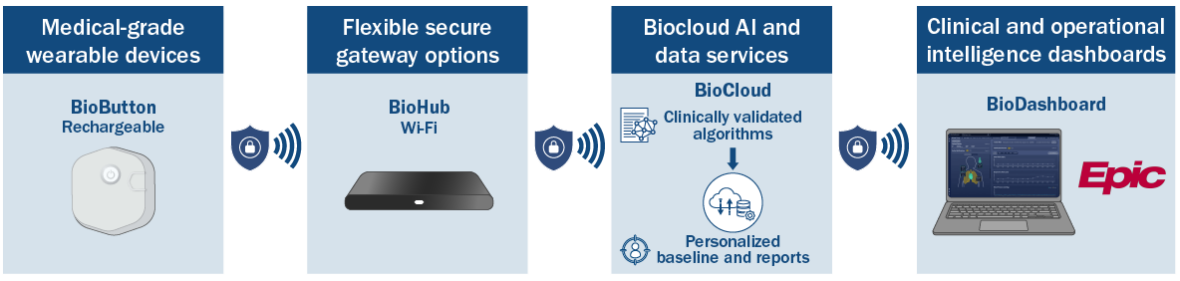
Data flow from the BioButton device through BioHub, BioCloud, and BioDashboard to Epic. AI: artificial intelligence.

Building on program planning, readiness efforts focused on 5 domains: room and BioHub setup, device and supply chain readiness, Epic and IT validation, unit training plans, and kickoff planning. Activities occurred at both system and unit levels (details are provided in Multimedia Appendix 3). Integration with Epic and the monitoring dashboard followed standard institutional IT and security protocols. IT preparations included configuring BioHub connectivity, enabling secure transmission of physiological data, and validating data flow into Epic flowsheets and early warning score logic. A summary of this pathway is shown in Figure 2.

Device and environmental preparations included stocking BioButton devices, labeling return bins, and completing room readiness surveys. Initial stock levels were based on recent admission volumes, with automated reordering through the supply chain. Workflows were established for device return and sanitation.

Kickoff planning finalized rollout sequencing and support allocation. The central monitoring team staffing within the VOC was expanded by reallocating staff after reducing telemetry use. VOC staff completed vendor-led training and competency demonstrations in alert interpretation, escalation protocols, and workflow integration.

#### Phase 4: Implementation and Optimization

Implementation emphasized 5 core activities: playbook rollout, staff training, go-live support, protocol refinement, and iterative feedback and optimization. Each unit followed an 8-week rollout guided by the playbook, with superuser and vendor support during the go-live phase.

Training followed a train-the-trainer model, with unit leaders and superusers trained first, followed by cascading training to frontline staff. All bedside nurses completed a mandatory learning management system module (100% completion system wide), which covered device application, alert interpretation, escalation protocols, and workflow integration. Adoption was reinforced through superuser rounding, vendor staff presence, and structured communication plans. Training activities were designed to build knowledge, confidence, and consistency among staff and are consistent with approaches described in the implementation science literature [21-23].

Go-live execution proceeded in staggered groups of 4 to 8 units, allowing overlap between training and operational transitions. Kickoff meetings, leadership communication plans, and coordinated supply chain activation supported each launch.

Protocol refinement was embedded throughout the rollout. Initial thresholds and workflows were iteratively adjusted based on frontline experience. VOC champions and superusers reinforced troubleshooting and informed subsequent launches. Sustainability was achieved by embedding CVSM into standard operations, including VOC monitoring workflows, Epic documentation, supply chain logistics, and updated learning management system training modules. Continued superuser engagement and institutional commitment to operational funding reinforced adoption. Full institutionalization of CVSM into existing structures ensured long-term ownership and positioned the program as a core element of HM’s broader digital care transformation strategy.

### Ethical Considerations

The Institutional Review Board of Houston Methodist Research Institute reviewed this initiative and deemed it *not human research*, as it represented a service redesign implemented as part of standard care.

## Implementation (Results)

### Coverage

Between April 2023 and February 2024, the program expanded from pilot units to enterprise-wide coverage across all 8 hospitals, encompassing the full adult, non-ICU inpatient population (approximately 2700 beds across the academic flagship, 6 community hospitals, and 1 LTAC facility).

### Rollout Approach and Timeline

The enterprise rollout followed a phased road map. Pilot implementation began on 4 units at the main hospital (2 internal medicine units, 1 neurosurgery unit, and 1 neurology unit). Four months later, deployment scaled through staggered 8-week waves of 4 to 8 units, with 3-week offsets to permit overlap between staff training and transitions, allowing the incorporation of iterative learning. This cadence enabled continuous optimization while maintaining operational continuity.

### Monitoring and Escalation Workflow

Five months after the pilot launch, HM introduced a standardized escalation protocol embedded in centralized monitoring workflows. When BioButton device–triggered alerts appeared on the VOC dashboard, a VOC technician performed a first-line review to add clinical content and either dismissed nonactionable events or escalated alerts to a VICU nurse. Approximately half of the alerts were dismissed at the VOC step, reducing alert burden for frontline staff. Illustrative case examples are provided in Multimedia Appendix 4.

The VICU nurse notified the bedside nurse or activated the clinical emergency response team when intervention was indicated. This tiered model concentrated clinical escalation on higher-value alerts. Data on clinical emergency response team activations during the study period have been reported previously in a related outcomes study [24].

### Outcomes and Post–Go-Live Protocol Refinement

Primary outcomes included system-wide scale-up, reliable Epic integration, and sustained monitoring. Device use exceeded 95%, training completion reached 100%, and approximately 50% of alerts were resolved at VOC review. Secondary outcomes included improved alert specificity, fewer patient refusals, increased staff acceptance, and operational efficiencies (nursing and PCA time savings). Alert thresholds were systematically reviewed with virtual medicine leadership in collaboration with frontline nursing staff, superusers, and the vendor. Baseline thresholds (HR 50-115 beats per minute, RR 11-28 breaths per minute, and skin temperature >36 °C) were operationalized with alerts stratified as single alerts (1 abnormal VS), double alerts (2 abnormalities, typically HR and RR), or triple alerts (3 abnormalities: HR, RR, and skin temperature). Three protocol changes improved the system-wide specificity.

Skin temperature was removed as an isolated escalation criterion and retained only for triple alerts. The RR lower limit was reduced from 11 to 8 breaths per minute to reduce clinically insignificant overnight alerts. The upper HR limit was increased from 115 to 120 beats per minute to reduce nonactionable alerts reported by community hospitals and the LTAC facility. Other parameters (eg, body position and activity) contributed to *low motion* alerts but were not primary triggers.

**Figure 3:**
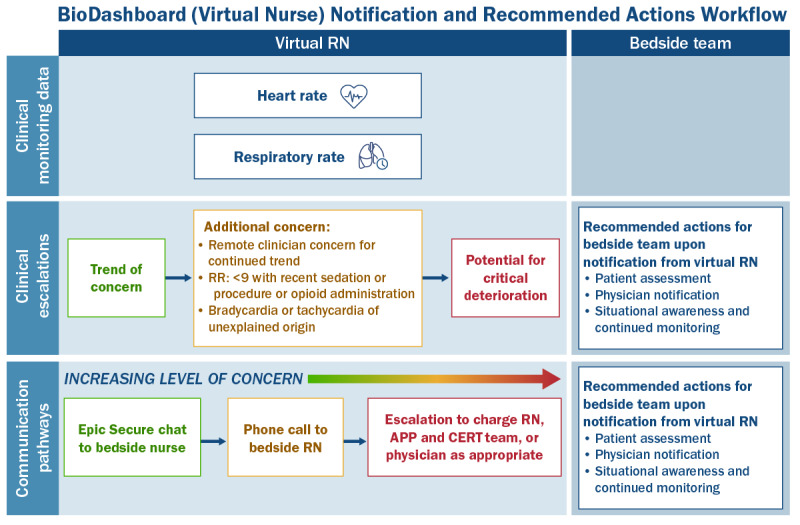
Escalation workflow for the BioButton device alerts with virtual operations center triage and bedside escalation. Bedside teams are responsible for all button management activities (ie, placement or removal, low battery, and no data). Patient data (from the BioIntelliSense button) will be transmitted into the Epic vital signs flowsheet, and BioDashboard can take up to 20 minutes to fully synchronize. APP: advanced practice provider; CERT: clinical emergency response team; RN: registered nurse; RR: respiratory rate.

**Figure 4:**
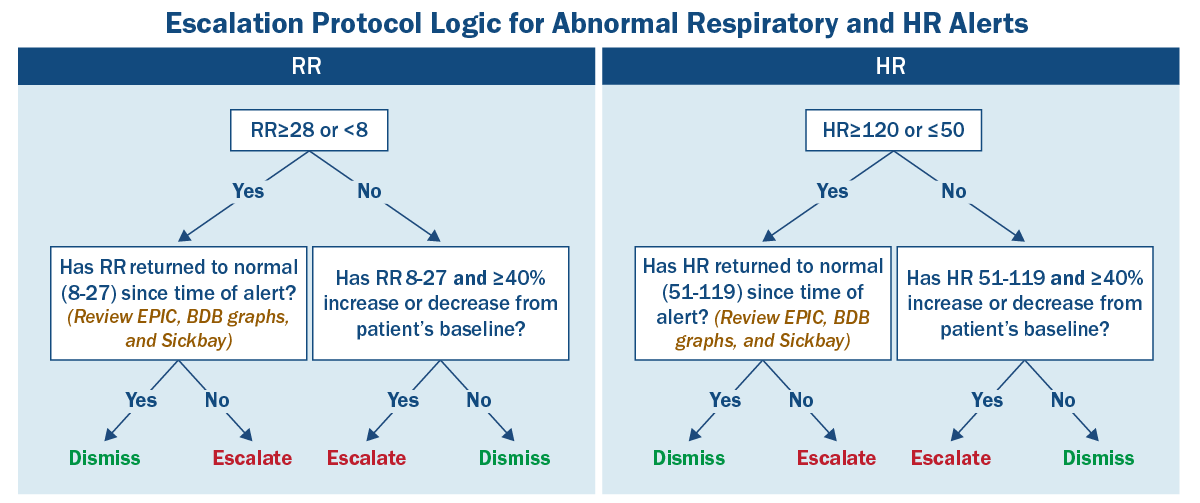
Escalation protocol for respiratory rate (RR) and heart rate (HR) alerts with system-wide thresholds. Final threshold for HR (50-120 bpm) and RR (8-27 bpm). Skin temperature has been removed from the escalation criteria. BDB: BioDashboard.

Collectively, these adjustments improved the balance between sensitivity and specificity, as reflected by observed reductions in nonactionable alerts and staff-reported decreases in false positives during enterprise rollout. The final escalation workflow and logic are depicted in Figures 3 and 4, with detailed initial and revised threshold values in Multimedia Appendix 5.

### Lessons Learned and Implementation Challenges

Technical and workflow challenges identified during the pilot informed the enterprise rollout. Barcode scanning difficulties (eg, poor label contrast) occasionally impeded device-to-patient matching; staff used device packaging barcodes and Workstations on Wheels carts as interim work-arounds, and the vendor subsequently improved barcode resolution. Rare *double HR* artifacts (concurrent S1 and S2 detections) produced nonactionable alerts and were resolved via algorithm updates.

Variation in device application sometimes caused skin irritation, which was addressed via staff reeducation and the use of adhesive remover spray. Early staff skepticism about using median CVSM values for documentation declined as experience accumulated, and staff perceived CVSM values to be accurate and consistent with routine bedside measurements; patient refusals decreased with standardized education. These mitigations and training reinforcements were propagated system wide during enterprise rollout.

As noted previously, unintended consequences, including minor skin irritation, initial device hesitancy, and early false positives, were observed and addressed through staff education, supplies, and vendor refinements; no significant harms were reported.

## Discussion

### Principal Findings

To our knowledge, this initiative represents the first large-scale deployment of wearable CVSM across a diverse, multihospital health system as part of a system-wide redesign initiative [17]. Our goal was to modernize inpatient VS practices by introducing continuous monitoring to support earlier detection, improve outcomes, and enhance both patient and clinician experience. Several units extended overnight VS intervals from every 4 hours to 6 to 8 hours, saving approximately 4 hours per nursing shift and allowing PCAs to focus on patient mobility and personal needs. Together with early clinical benefits, these efficiencies strengthened adoption and staff buy-in.

Weller et al [17] evaluated the same FDA-cleared device in 11,977 patients across urban and rural hospitals, with initial ICU use before expansion to non-ICU settings. They reported low RR alarms most frequently, while in our implementation, elevated RR emerged as the most common alert, underscoring RR’s role as a sensitive marker of cardiopulmonary function. This difference likely reflects patient mix, as sedation in ICU patients tends to lower RRs, while elevated RRs in ward patients more often indicate deterioration.

A key distinction of this program was its breadth and scope. Unlike previous efforts limited to ICUs, daytime hours, or narrow diagnostic groups, this program encompassed all adult non-ICU inpatients, deployed continuously (24×7) across the flagship academic hospital, 6 community hospitals, and 1 LTAC facility. This design addressed gaps in every 4-hour VS collection, enabled earlier detection across diverse patient populations, and supported workflow redesign amid nursing staff shortages.

Device selection prioritized accurate RR measurement, given its strong predictive value. Although the BioButton devices do not measure blood pressure or SpO_2_, they remained available through existing bedside monitoring. Continuous SpO_2_ monitoring was avoided to minimize alarm burden. The device offered unobtrusive, trended monitoring without disturbing patients’ rest while capturing key physiological parameters for early detection. The final escalation protocol’s emphasis on HR and RR reflected clinical prioritization and not device limitation. Although formal satisfaction and quality metrics are forthcoming, early stakeholder feedback suggests that reduced overnight disruptions improved sleep quality, potentially enhancing patient satisfaction and reducing overnight nursing workload. Prospective studies are needed to evaluate long-term effects on clinical outcomes, alert trends, workflow efficiency, and satisfaction, including patient-reported outcomes.

### Limitations

This report has several limitations. No formal satisfaction survey was conducted, and estimates of time savings were based on staff feedback. BioButton device–derived VSs were not independently validated, as accuracy testing of the FDA-cleared device was beyond the scope of this work. The escalation protocol was intentionally delayed in pilot units; therefore, early data reflect monitoring without active escalation, with finalized protocols introduced later and applied retrospectively. Although guided by an internal business case projecting nursing and PCA time savings, no formal health technology assessment was conducted.

Generalizability may be limited by HM’s centralized monitoring model; other health systems may face different challenges. Observed alert trends may also differ across settings, as illustrated by differences between ICU-focused studies and our broad non-ICU deployment. Outcome measures, such as clinical impact, workflow efficiency, and adoption, were not prospectively defined, constraining evaluation but informing priorities for future analyses.

Several transferable lessons emerged: (1) structured change management with engagement, champions, and feedback loops supported adoption; (2) phased rollout with iterative refinements reduced risk and enabled optimization; (3) centralized monitoring enabled 24×7 oversight, while hospitals without such infrastructure could adapt using telemetry staff, rapid response teams, or external partners; and (4) alignment of CVSM with institutional priorities secured leadership support and momentum. Table 1 summarizes key lessons and success factors to guide future implementers in scaling similar initiatives.

**Table 1:** Key lessons and success factors from system-wide CVSMa implementation.

Domain	Challenges encountered	Lessons learned and success factors
Leadership and Governance	Balancing innovation with standardization across 8 hospitals	Early executive sponsorship and system-wide alignment maintained focus, accountability, and resource continuity.
Implementation Approach	Scaling across multiple hospitals while managing operational risk	Staggered enterprise rollout using an 8-week implementation playbook enabled iterative refinement and risk mitigation before full scale-up.
Workflow Integration	Incorporating CVSM data into existing Epic processes	Integration into Epic flowsheets and early warning scores streamlined workflow and improved adoption.
Technology and Infrastructure	Interoperability issues and alert fatigue	Centralized monitoring filtered ~50% of alerts before bedside escalation; iterative threshold refinements improved specificity and reduced alert fatigue.
Training and Adoption	Staff skepticism and learning curve	Superuser model, LMS^b^ training, and vendor support during go-live built confidence and sustained engagement.
Change Management	Variation in readiness and engagement across sites	Phased rollout enabled iterative optimization and local adaptation while maintaining operational continuity.
Data Governance	Managing HIPAA^c^ compliance and device data retention	Preimplementation IT security review ensured compliance with institutional privacy and security standards.
Sustainability	Maintaining engagement and support after implementation	Integration into standard operations, funding streams, and training infrastructure supported long-term sustainability.
Cultural Readiness	Early staff skepticism about device accuracy and value	Confidence increased with experience and demonstrated accuracy, reinforcing trust and normalization of continuous monitoring.

^a^CVSM: continuous vital sign monitoring.

^b^LMS: learning management system.

^c^HIPAA: Health Insurance Portability and Accountability Act.

### Conclusions and Future Implications

This initiative demonstrates the feasibility and operational value of scaling CVSM across a large, diverse health system. Embedding continuous monitoring into standard workflows can improve patient safety, enhance staff efficiency, and build infrastructure for digitally enabled care models.

Future work should rigorously assess clinical outcomes, economic impact, and patient-reported measures while exploring application in postacute and ambulatory settings. Although growing evidence supports continuous monitoring, prospective randomized trials are still needed to confirm clinical and economic benefits. As health systems address workforce shortages and rising patient acuity, wearable monitoring technologies, such as CVSM, could serve as core infrastructure for safer, more efficient, and patient-centered care.

## Appendix

Multimedia Appendix 1 Physiological parameters measured by the BioButton device, measurement methods, and clinical use cases.

Multimedia Appendix 2 Roles and responsibilities of vendor and health system stakeholders in the multidisciplinary implementation team for continuous vital sign monitoring.

Multimedia Appendix 3 System-level and unit-level readiness activities by operational domain for continuous vital sign monitoring implementation.

Multimedia Appendix 4 Illustrative case examples of early detection and intervention.

Multimedia Appendix 5 Initial and revised alarm thresholds with rationale for revisions.

Multimedia Appendix 6 iCHECK-DH: Guidelines and Checklist for the Reporting on Digital Health Implementations.

## Abbreviations

CVSM: continuous vital sign monitoring
FDA: Food and Drug Administration
HM: Houston Methodist
HR: heart rate
ICU: intensive care unit
LTAC: long-term acute care
PCA: patient care assistant
RPM: remote patient monitoring
RR: respiratory rate
SpO2: oxygen saturation
VICU: virtual intensive care unit
VOC: virtual operations center
VS: vital sign

## Data Availability

The datasets generated or analyzed during this study are not publicly available due to institutional privacy policies.
