# Supplementary material for: Scaling Wireless Continuous Vital Sign Monitoring Across an 8-Hospital Health System: Digital Health Implementation Report
Source: JMIR Med Inform. 2026 Jan 26;14:e78216. doi: 10.2196/78216 (PMC12887559; doi:10.2196/78216)
Supplement: Multimedia Appendix 1 [file medinform_v14i1e78216_app1.docx]

| **Vital Sign / Parameter** | **How Measured** | **Clinical Use Case** |
| --- | --- | --- |
| Skin Temperature | Continuous skin sensor | Fever detection, infection monitoring, circadian trends |
| Respiratory Rate (RR) | Derived from chest wall movement (impedance/accelerometry) | Early detection of deterioration, respiratory compromise |
| Heart Rate (HR) | Derived from impedance/accelerometry | Monitoring cardiac status, stress, deterioration |
| Body Position & Orientation | Accelerometer-based (supine, prone, sitting, standing, fall orientation) | Fall risk assessment, mobility tracking, adherence to positioning |
| Activity / Step Count | Accelerometer | Recovery monitoring, activity tolerance |
| Activity Level (Low/Med/High) | Accelerometer + activity classification algorithms | Functional status, rehabilitation progress, daily activity trends |
| Activity Duration | Derived from accelerometer + activity classification | Monitoring sedentary vs. active periods, sleep hygiene support |
| Cough Frequency | Acoustic/vibration algorithms | Respiratory illness monitoring, COVID-19/post-viral follow-up |
| Sleep / Rest-Wake Cycles | Activity + physiologic signals | Sleep quality, recovery patterns, circadian rhythm assessment |
| Device/Wear-Time Indicators | Sensor integrity, skin contact detection | Ensuring data quality, identifying non-wear periods, guiding staff interventions |
