# Supplementary material for: Scaling Wireless Continuous Vital Sign Monitoring Across an 8-Hospital Health System: Digital Health Implementation Report
Source: JMIR Med Inform. 2026 Jan 26;14:e78216. doi: 10.2196/78216 (PMC12887559; doi:10.2196/78216)
Supplement: Multimedia Appendix 2 [file medinform_v14i1e78216_app2.docx]

| **Role** | **Vendor Responsibilities** | **Health System Responsibilities** |
| --- | --- | --- |
| ***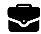 Strategic Oversight*** | | |
| Executive Sponsor(s) | Provide escalation assistance as needed | Motivate internal teams, identify project leads, manage issues, and oversee coordination |
| Project Manager | Develop project plan and schedule and manage risks | Track deliverables, coordinating communications and risks |
| ***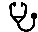 Clinical Leadership*** | | |
| Physician and Nursing Leads | Lead workflow discussions, define success metrics, participate in testing | Confirm project scope, participate in current and future-state workflow design, confirm alerts and response plan, lead training, and review metrics post-go live |
| Command Center Med/Nurse Lead | Provide alert examples and training | Determine alert response protocols and escalation pathways, train VOC personnel, and ensure 24/7 monitoring coverage |
| 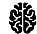 ***Operations, Logistics, and Support*** | | |
| Operational Lead | Manage logistics and shipping | Coordinate procurement and return/reprocessing workflows and internal operations to manage devices |
| Training & Education Lead | Develop and deliver training, provide materials and user guides | Coordinate with vendor on training materials and sessions, serve as super user |
| Customer Care | Provide support post go-live including troubleshooting and Return Merchandise Authorization (RMA) support | Submit support tickets and RMAs |
| Data Science | Share dashboards (device activation, compliance, and alert data) conduct case reviews | Establish data extraction frequency and provide data and/or analysis to support metric evaluation |
| **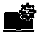 Technology & Integration** | | |
| IT Integration Lead | Provide integration requirements and assist with testing | Assign resources, oversee integration planning and testing |
| Interface Engineer | Plan, build, and test interfaces | Complete and validate interface builds |
| Network Engineer | Support VPN and network access testing | Install BioHubs, configure network access |
| EMR Application Analyst | Specify clinical data integration and alerting specifications for testing | Build Epic order entry and alert functionality |
| Biomedical Engineer | Test BioHub installation | Identify BioHub locations, support setup and testing |
