# Supplementary material for: Scaling Wireless Continuous Vital Sign Monitoring Across an 8-Hospital Health System: Digital Health Implementation Report
Source: JMIR Med Inform. 2026 Jan 26;14:e78216. doi: 10.2196/78216 (PMC12887559; doi:10.2196/78216)
Supplement: Multimedia Appendix 3 [file medinform_v14i1e78216_app3.docx]

| **Operational Domain** | **System-Level Activities** | **Unit-Level Activities** |
| --- | --- | --- |
| 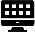 **IT & Integration** | Configured Epic filters for new units, updated lookup tables, and set up BioDashboard census. Validated end-to-end data flow between BioIntelliSense and Houston Methodist IT systems. | Enabled Epic flowsheets, confirmed BioIntelliSense messaging, and configured access. Conducted room readiness surveys to confirm adequate power sources and hub installation requirements. |
|  | *8 months prior to implementation* | *2 months prior to go-live* |
| **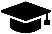 Training & Education** | Delivered bedside staff training and completed end-to-end validation of technical components. Refreshed LMS modules and enrolled users across all units. | Trained unit staff and finalized unit-specific training plans and LMS updates. |
|  | *6 months prior to implementation* | *2 weeks prior to go-live and ongoing during implementation* |
| **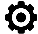 Device & Environmental Setup** | Monitored real-time device data flow, BioHub performance, and support tickets with vendor and IT teams. | O Ordered, installed, and labeled BioHubs and BioButton holders in patient rooms; labeled return boxes and set up collection bins. Placed return bins in dirty utility rooms to support device sanitation workflows. |
|  | *3-6 months prior to implementation* | *2 months prior to go-live* |
| **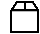 Supply Chain & Logistics** | Stocked devices and accesories, configured resupply and sanitation workflows, and established return logistics. Estimated initial stock levels based on recent admission volumes, with a margin of error to prevent shortages. Implemented automated reordering triggered when bin weights dropped below designated par levels. | Oriented operational leads to logistics and return/reprocessing workflows. |
|  | *5 months prior to implementation* | *2 weeks prior to go-live* |
| **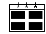 Planning & Go- Live Support** | Defined unit rollout sequence, allocated support resources, and conducted kickoff meetings. Finalized workflow planning and VOC alert coordination. | Moved IT components to production; deployed devices with bedside staff. Vendor provided onsite support. Handoff to operational support teams. |
|  | *2–6 months prior to implementation* | *Weeks 6 to 8 of unit go-live* |

*Technical Integration Summary*

As part of system readiness, IT teams completed the detailed technical integration required for continuous monitoring. This included configuration of BioHub gateway connectivity, secure transmission of BioButton data to the vendor cloud, encryption at rest and in transit, processing of hourly median values, and integration of physiologic parameters into Epic flowsheets and early warning score logic via HL7/FHIR interfaces. Device-to-patient matching occurred through Epic Rover barcode scanning, with manual entry as a fallback. Missing parameters in EWS calculations defaulted to the most recent EMR values per Epic logic. Integration underwent institutional IT security review, technical validation testing, and end-user workflow testing. Data were exchanged as discrete physiologic values but were not mapped to standard vocabularies such as LOINC or SNOMED CT.
