# Supplementary material for: Scaling Wireless Continuous Vital Sign Monitoring Across an 8-Hospital Health System: Digital Health Implementation Report
Source: JMIR Med Inform. 2026 Jan 26;14:e78216. doi: 10.2196/78216 (PMC12887559; doi:10.2196/78216)
Supplement: Multimedia Appendix 4 [file medinform_v14i1e78216_app4.docx]

*Summary Table*

| **Case** | **Profile** | **Alert (parameter)** | **Intervention** | **Outcome** |
| --- | --- | --- | --- | --- |
| 1. Heart failure & CKD with wide-complex tachycardia | 65-year-old male with multiple comorbidities, including chronic kidney disease and congestive heart failure, admitted for suspected sepsis | HR: Wide-complex tachycardia (160s) on first hospital night | VOC escalated to CERT | CERT initiated sepsis protocol; patient transferred to ICU; positive recovery trajectory |
| 1. PTEN hamartoma tumor syndrome postop | 47-year-old patient with hereditary cancer syndrome, recovering from total colectomy | HR + RR: Tachycardia and tachypnea | VOC escalated for urgent bedside evaluation and rapid surgical team involvement | Returned to OR; subsequently admitted to ICU for stabilization |
| 1. Overnight respiratory distress | Adult patient with heart failure and COPD | RR + HR: Respiratory distress with tachycardia on first hospital night | VOC escalated to CERT | Early intervention prevented further deterioration; timely escalation to ICU |

*Narrative Vignettes*

1. *Case 1: Heart failure and CKD with wide-complex tachycardia:* A 65-year-old male with multiple comorbidities, including chronic kidney disease and congestive heart failure, was admitted for suspected sepsis. On the first hospital night, the VOC team received an abnormal HR alert for wide-complex tachycardia (160s). The alert was escalated to CERT, who initiated the sepsis protocol and transferred the patient to the ICU, resulting in a positive recovery trajectory.
2. *Case 2: PTEN hamartoma tumor syndrome following colectomy:* A 47-year-old patient with PTEN hamartoma tumor syndrome, recovering from total colectomy, triggered HR and RR alerts (tachycardia and tachypnea). The VOC escalated for urgent bedside evaluation and surgical team involvement. The patient was returned to the operating room and subsequently admitted to the ICU for stabilization.
3. *Case 3: Overnight respiratory distress:* An adult patient with a history of heart failure and COPD developed RR and HR alerts (respiratory distress with tachycardia) during the first hospital night. The VOC escalated to CERT, leading to rapid bedside evaluation. Early intervention prevented further deterioration and ensured timely ICU transfer.
