# Supplementary material for: Scaling Wireless Continuous Vital Sign Monitoring Across an 8-Hospital Health System: Digital Health Implementation Report
Source: JMIR Med Inform. 2026 Jan 26;14:e78216. doi: 10.2196/78216 (PMC12887559; doi:10.2196/78216)
Supplement: Multimedia Appendix 5 [file medinform_v14i1e78216_app5.docx]

| **Vital Sign/Parameter** | **Initial Thresholds** | **Final Thresholds** | **Rationale for Revision** |
| --- | --- | --- | --- |
| Heart rate (HR) | 50–115 bpm | 50–120 bpm | Raised upper limit to reduce non-actionable alerts (~5 months post-enterprise launch) |
| Respiratory rate (RR) | 11–28 breaths/min | 8–27 breaths/min | Lower limit reduced after excessive clinically insignificant overnight alerts (~3 months post-enterprise launch) |
| Skin temperature | >36 °C | >36 °C for triple alerts only | Retained for triple alerts but removed from single/double pathways due to poor correlation with core body temperature and frequent false alarms (at enterprise launch) |
