# Supplementary material for: Scaling Wireless Continuous Vital Sign Monitoring Across an 8-Hospital Health System: Digital Health Implementation Report
Source: JMIR Med Inform. 2026 Jan 26;14:e78216. doi: 10.2196/78216 (PMC12887559; doi:10.2196/78216)
Supplement: Multimedia Appendix 6 [file medinform_v14i1e78216_app6.pdf]

Alignment of Houston Methodist Continuous Vital Signs Monitoring (CVSM) Implementation Paper to JMIR's iCHECK-DH: Guidelines and Checklist for the Reporting on Digital Health Implementations

SECTION	ITEM		DESCRIPTION	TEXT EXTRACTED FROM MANUSCRIPT (WITH SUPPLEMENTARY CLARIFICATIONS IN *ITALICS*)<br>**Note**: All text in this column is drawn verbatim from the final clean manuscript. Supplementary clarifications are provided in *italics* solely for checklist readability; no new content has been introduced.
**TITLE**	1	Title (M[1])	Identification as an implementation report, and description of the implementation in the title and/or keywords	"Scaling Wireless Continuous Vital Signs Monitoring Across an Eight-Hospital Health System: A Digital Health Implementation Report"
**ABSTRACT**	2	Abstract (M)	Provide a summary of the key elements of the implementation report, including a description of the implementation strategy, the intervention, defining the key elements of the implementation and health outcomes and specify the key KPIs/Outputs. We recommend describing the main aspects of the research in the following order: Background - Objectives - Methods - Implementation (Results) - Conclusions - (Optional: Trial Registration).	"**Background**: Frequent vital signs (VS) monitoring is central to inpatient safety but is traditionally measured manually every four hours, a century-old practice that can miss early deterioration, disrupt patient sleep, and impose a heavy documentation burden on nursing staff. Continuous vital signs monitoring (CVSM) using wearable remote patient monitoring (RPM) devices enables near real-time, high-frequency VS measurement while reducing manual workload and preserving patient rest.<br>**Objectives**: This implementation report describes the large-scale implementation of CVSM across an eight-hospital health system. The initiative aimed to: (1) enhance earlier detection of patient deterioration through continuous, algorithm-driven monitoring; (2) improve nursing workflow efficiency by reducing reliance on manual VS checks; and (3) minimize nighttime disruptions to support patient rest and recovery.<br>**Methods**: The program was designed for system-wide scalability and executed from 2022 to 2024 using a four-phase framework: strategic program design, program planning, go-live preparation, and implementation and optimization. The FDA-cleared wearable device continuously measured heart rate, respiratory rate, and skin temperature, with integration into Epic and 24/7 oversight through a centralized Virtual Operations Center (VOC). Rollout followed a staggered playbook across ~2,700 adult non-ICU beds, supported by leadership engagement, supply chain readiness, training, and phased superuser-led adoption.<br>**Implementation (Results):** All eight hospitals achieved full deployment between April 2023 and February 2024, with >95% device utilization rates and 100% nursing staff training completion. A standardized escalation workflow filtered ~50% of alerts at the VOC review step, substantially reducing frontline alert burden. Operational refinements included revised HR and RR thresholds and removal of temperature as a single alert trigger. Several units extended overnight manual VS intervals from every four to every six–eight hours, with staff estimating ~4 hours saved per nursing shift. Patient care assistants redirected time toward mobility and personal needs, while staff reported growing confidence in device accuracy.<br>**Conclusions**: This initiative represents the first system-wide deployment of CVSM across a diverse, multi-hospital health system. Success was enabled by early strategic alignment, phased rollout, robust IT and monitoring infrastructure, and iterative optimization. The program demonstrates the feasibility of embedding CVSM into routine inpatient care to improve efficiency and patient experience. Transferable strategies, including phased rollouts, centralized monitoring, and structured change management, may inform other health systems pursuing digital vital signs redesign. Future work should rigorously evaluate impacts on patient outcomes, cost-effectiveness, and applicability to post-acute and ambulatory care<br>**Keywords**: Continuous Vital Signs Monitoring, Remote Patient Monitoring, Wearable Devices, Hospital Workflow Redesign, Patient Safety, Digital Health Implementation, Implementation Science"

[1] M: Mandatory item

Alignment of Houston Methodist Continuous Vital Signs Monitoring (CVSM) Implementation Paper to JMIR's iCHECK-DH: Guidelines and Checklist for the Reporting on Digital Health Implementations

SECTION	ITEM		DESCRIPTION	TEXT EXTRACTED FROM MANUSCRIPT (WITH SUPPLEMENTARY CLARIFICATIONS IN *ITALICS*)<br>**Note**: All text in this column is drawn verbatim from the final clean manuscript. Supplementary clarifications are provided in *italics* solely for checklist readability; no new content has been introduced.
**INTRODUCTION**	3	Context (M)	Describe the geographical areas, organizations, target populations and implementation context. Consider social, cultural, economic, political, health care and organizational barriers, infrastructures and facilitators that may influence implementation elsewhere. Explicitly highlight whether a national digital health strategy exists and whether implementation is aligned with the strategy.<br><br>Describe the stage of the implementation (Developing or Adapting Solution / Piloting and Evidence generation / Package and Advocacy /Acceleration / Deploying / Scaling up / Hand over or Complete).[2]	"This implementation occurred within Houston Methodist (HM), an eight-hospital health system in Texas encompassing Houston Methodist Hospital (HMH), a quaternary care academic flagship; six community hospitals; and one long-term acute care facility serving the greater Houston area, together serving a diverse adult population of more than 140,000 annual admissions and 2 million outpatient visits.<br><br>During the COVID-19 pandemic, HM established a centralized Virtual Operations Center (VOC) with a Virtual Intensive Care Unit (VICU) program providing 24/7 monitoring for approximately 370 ICU patients. This infrastructure created favorable conditions for expanding remote patient monitoring (RPM) capabilities and ultimately informed development of the system-wide CVSM program. This enterprise-wide CVSM program represents a form of inpatient remote monitoring but differs from traditional RPM in its continuous, system-integrated design.<br><br>In 2022, the health system launched a redesign of inpatient vital signs (VS) practices, with this report detailing the program's scaling up and deployment, designed from the outset for system-wide implementation."
	4	Problem statement (M)	Description of the health care or public health problem, challenge, or deficiency that the implementation aims to address. (If applicable, include a reference to the 'health system challenge' of the WHO Classification of Digital Health Interventions[3] in the description)	"Frequent monitoring of vital signs (VS)—heart rate (HR), respiratory rate (RR), temperature, blood pressure, and oxygen saturation (SpO_2)—is a cornerstone of inpatient safety. On general hospital floors, VS are still collected manually every four hours, a century-old practice that does not account for patient acuity. This approach risks missed deterioration between checks, places heavy time demands on nursing staff, and disrupts sleep with physiologic consequences. Abnormal VS are well-established predictors of ICU transfer and in-hospital mortality, and CVSM, enabled by wearable RPM devices, now allows for unobtrusive, high-frequency VS measurement. This work directly addresses health system challenges of limited access to timely clinical data and variable adherence to evidence-based monitoring practices, both of which undermine patient safety and workflow efficiency. However, evidence on broad system-wide implementation across diverse hospital settings is limited."<br><br>*This initiative addresses the WHO health system challenges of limited access to timely clinical data and variable adherence to evidence-based monitoring standards, both of which undermine patient safety and workflow efficiency.*
	5	Similar Interventions (M)	Mention whether this implementation was inspired by another existing one, and if so, what is the added value of your intervention, if any, compared to the initial one? And what, if anything, has been done differently?	"Prior CVSM implementations have typically been limited to ICUs, single hospitals, or narrow patient groups, demonstrating feasibility but limiting generalizability. Our initiative differs by extending CVSM across all adult non-ICU inpatients in an eight-hospital system, integrated with Epic and supported by centralized monitoring. Unlike earlier efforts, it was designed from the outset for system-wide scale, with early adopter units refining

[2] *Stages adapted from WHO Digital Health Atlas*

[3] https://apps.who.int/iris/bitstream/handle/10665/260480/WHO-RHR-18.06-eng.pdf?sequence=1&isAllowed=y

Alignment of Houston Methodist Continuous Vital Signs Monitoring (CVSM) Implementation Paper to JMIR's iCHECK-DH: Guidelines and Checklist for the Reporting on Digital Health Implementations

SECTION	ITEM		DESCRIPTION	TEXT EXTRACTED FROM MANUSCRIPT (WITH SUPPLEMENTARY CLARIFICATIONS IN *ITALICS*)<br>**Note**: All text in this column is drawn verbatim from the final clean manuscript. Supplementary clarifications are provided in *italics* solely for checklist readability; no new content has been introduced.
				workflows to inform broader rollout. By embedding CVSM into routine inpatient care across diverse hospital contexts, this program provides new insights into scaling digital vital signs monitoring at enterprise level."<br><br>*The added value of this initiative is its system-wide scope, Epic integration, and centralized Virtual Operations Center triage model, which extend beyond the feasibility focus of prior single-site implementations.*
**METHODS**	**6**	Aims and Objectives (M)	Describe the main objectives and the overall aim of the implementation. Describe how these will be measured using predefined primary and secondary outcome(s) and key performance indicators for this implementation and the expected intervention(s).<br>*For example: indicators or proxy-indicators measuring direct health outcomes (e.g., HbA1c for diabetic patients); Key Performance IndiWHOcators (e.g., number of users, number of users that are properly trained, user satisfaction); Indicator assessing a particular process (e.g., administrative time for patient admission);*<br>(If there was no evaluation, provide detailed explanation for reasoning)	"The overall aim of this initiative was to conduct a comprehensive redesign of inpatient vital signs (VS) measurement practices to better support patient safety, experience, and staff efficiency through implementation of CVSM. The intervention objectives were to detect deterioration earlier, reduce nighttime disruptions to promote patient rest and recovery, and improve workflow efficiency by decreasing reliance on manual VS checks.<br><br>The implementation objectives focused on successful rollout and adoption, assessed through device utilization, staff training completion, and reductions in manual VS collection, with qualitative observations on staff and patient acceptance. No direct health outcome evaluation was conducted, as this quality improvement initiative focused on service redesign and operational efficiency**."**<br><br>*Primary outcomes included full enterprise rollout, Epic integration, and sustained VOC monitoring. Secondary outcomes included device utilization, 100% staff training completion, reduced manual VS collection, and qualitative feedback on staff/patient acceptance. Direct health outcomes (e.g., ICU transfer rates, mortality) were not assessed, as this was a service redesign initiative.*
	7	Blueprint summary (M)	Describe the design and key features of the intervention and key points of the implementation strategy and roadmap.	"The implementation strategy was structured around a four-phase framework designed for system-wide scalability, and the roadmap in Figure 1 reflects the observed implementation timeline across the health system."<br><br>*The four phases included: (1) Strategic Program Design, defining goals and target populations; (2) Program Planning, including vendor selection, workflow redesign, and business case development; (3) Go-Live Preparation, with IT integration, training, and supply chain readiness; and (4) Implementation and Optimization, with staggered unit rollouts, superuser training, and iterative protocol refinements.*
	8	Technical Design (M)	Reasons for developing or choosing this tool. Does it combine several tools? Provide a brief description of the tool(s) (functionality and architecture) and how it fits into the health enterprise architecture and investment roadmap (if applicable). Indicate whether the solution is based on an existing solution or has been	"A wearable patch device (BioButton®, BioIntelliSense, Golden, CO, USA) was selected as the FDA-cleared option that best met criteria for accuracy, usability, and Epic integration. The medical-grade, one-inch adhesive device is applied to the upper left chest and powered by a battery with a 7-day lifespan in acute mode and 16 days in post-acute mode. It continuously collects heart rate (HR), respiratory rate (RR), and skin temperature, as well as body position, activity level (low, medium, or high), cough frequency, and sleep–wake cycles. Device selection prioritized accurate RR measurement, given its strong predictive value for

SECTION		ITEM	DESCRIPTION	TEXT EXTRACTED FROM MANUSCRIPT (WITH SUPPLEMENTARY CLARIFICATIONS IN *ITALICS*)<br>**Note**: All text in this column is drawn verbatim from the final clean manuscript. Supplementary clarifications are provided in *italics* solely for checklist readability; no new content has been introduced.
			developed or purchased specifically for this intervention.<br><br>Describe the type of technology used (e.g., AI applications), license of the technology (open source, free, commercial, IP ownership etc.), include code documentation (if available), link to the application, link to wiki or project website.	deterioration. Additional selection factors included FDA clearance, multiparameter monitoring capability, battery life, patient comfort, and seamless Epic EMR integration.<br>The device does not measure oxygen saturation or blood pressure; these were considered acceptable omissions since both are already available through existing bedside monitoring and intermittent vital sign collection. Moreover, continuous SpO_2 monitoring typically requires a finger probe or tethered sensor that can disrupt rest and increase alarm burden, whereas the BioButton enabled unobtrusive, trended monitoring without disturbing sleep. Details of all physiologic parameters are provided in Multimedia Appendix 1. The program was approved as part of routine service delivery, with no additional costs to patients."<br><br>*The BioButton® is a commercial, FDA-cleared medical device owned by BioIntelliSense. Intellectual property for the device/software remains with BioIntelliSense, while Houston Methodist owns the workflows, data integration, and operational model.*
	9	Target (M)	The target refers to the focus or recipient of the intervention. It is the specific person, group, system, or problem that the intervention aims to change or improve. The characteristics of the targeted "site(s)" (locations, staff, resources, etc.) for implementation and any eligibility criteria. The population targeted by the intervention and any eligibility criteria.	"The primary use case was continuous monitoring of all hospitalized, non-ICU patients to fill the safety gap created by routine vital sign checks performed every four hours. Program goals included earlier detection of deterioration (particularly preventing unplanned ICU transfers), reducing overnight patient disruptions, and supporting workflow redesign in anticipation of projected nursing shortages. Patient eligibility was intentionally broad: CVSM was applied primarily to general adult medical-surgical inpatients, with no additional restrictions beyond exclusion of ICU patients. Implementation sites included the academic flagship, six community hospitals, and one long-term acute care facility, with monitoring supported by bedside nurses, unit superusers, and the centralized Virtual Operations Center (VOC)."<br><br>*Targeted recipients were adult, non-ICU inpatients across ~2,700 beds, supported by bedside staff, superusers, and centralized monitoring teams. The intervention aimed to address gaps in safety, workflow efficiency, and patient experience at the enterprise health system level.*
	10	Data (M)	Describe the data governance, including life cycle (collection, processing, storage, , sharing, suppression), the data ownership (mention whether patients actually have access to the data), data protection measures, confidential use of routine data, expected level of data integration, data for research, cross-border data agreement, if any, the applicable legal framework, and how the project complies with it. Data consent: Has patient consent been obtained? Describe the approach to data protection and cybersecurity (e.g. security by design, privacy by design, etc.) and where the	"Data governance measures ensured that all device data were encrypted in transit and at rest, stored in the HIPAA-compliant BioCloud (U.S.-based cloud hosting), and transferred hourly into Epic flowsheets for clinical documentation. As an FDA-cleared device, BioButton data were accepted for clinical use without independent validation and were not used for research. Data were collected continuously and processed into hourly median values; raw device data were discarded after transfer in accordance with institutional retention policies. Patients did not have direct access and did not need to consent as monitoring was implemented as standard of care. IT security teams validated compliance with HIPAA through a pre-implementation security review before go-live."<br><br>***Ownership**: BioIntelliSense retains intellectual property for the device/software, while Houston Methodist owns workflows, integration, and operational model.*<br>***Consent**: No patient consent was required because monitoring was implemented as standard of care.*

SECTION		ITEM	DESCRIPTION	TEXT EXTRACTED FROM MANUSCRIPT (WITH SUPPLEMENTARY CLARIFICATIONS IN *ITALICS*)<br>**Note**: All text in this column is drawn verbatim from the final clean manuscript. Supplementary clarifications are provided in *italics* solely for checklist readability; no new content has been introduced.
			data is hosted. (e.g., in-country, cloud based, hybrid model etc.). Describe, if applicable, the government preferences in terms of data policies.	***Legal framework**: Data protections were aligned with U.S. HIPAA regulations; no cross-border data agreements were involved.*
	11	Interoperability (M)	Describe the interfaces (what other systems does the tool connect to) and the standards that were used (which specific ones and rationale of choice) (e.g., semantic ontologies such ICD as SNOMED, LOINC or technical standards such as HL7 FHIR, etc.).	"Interoperability was achieved through HL7/FHIR interfaces, aligned with Epic's architecture and national standards. These enabled CVSM data to appear in Epic flowsheets alongside traditional vital signs and integrate into early warning score systems, ensuring both technical and workflow interoperability. Data were exchanged as discrete physiologic parameters but were not mapped to semantic ontologies such as LOINC or SNOMED CT."<br><br>*The system architecture pathway was: BioButton® → BioHub® Wi-Fi gateways → BioCloud™ (secure analytics platform) → BioDashboard™ → Epic EMR flowsheets and early warning scores, ensuring end-to-end data integration.*
	12	Participating entities (M)	Describe the implementing organization(s): Type of organisation(s), mission, leadership, vision, etc.<br>Government involvement: Describe whether the government was involved in the implementation, at what level and at what stage(s).<br>Partners: Describe all partners (organisations) and their role in the implementation.<br>Funders: List all actors and stakeholders who have funded or invested in the development of the implementation (if different from the implementation, e.g. using an existing digital health intervention). Indicate their level of involvement in terms of funding.<br>Mention which entity will own the final product and intellectual property after the implementation phase.	"Participating entities included Houston Methodist, a quaternary academic health system whose mission emphasizes patient-centered, digitally enabled care. Houston Methodist led strategy, workflows, monitoring staff, and supply chain integration. BioIntelliSense, a medical device company specializing in continuous physiologic monitoring, provided devices, hubs, dashboard development, and technical support. The Virtual Operations Center (VOC) delivered continuous monitoring, while hospital leaders designated champions, bedside nurses served as superusers, and IT, operations, and supply chain staff supported infrastructure and logistics.<br><br>The program was funded internally by Houston Methodist as part of service redesign; no government funding or involvement occurred. Intellectual property for the BioButton device and software remains with BioIntelliSense, while Houston Methodist owns the workflows, data integration, and operational model."<br><br>***Implementing organization**: Houston Methodist, a large, non-profit, faith-based health system with a mission to deliver patient-centered, digitally enabled care.*<br>***Partners & roles**: Houston Methodist (implementation lead), BioIntelliSense (device, hubs, technical support), VOC (24/7 centralized monitoring), hospital/unit leaders (champions, superusers), and IT/operations/supply chain teams (infrastructure & logistics).*<br>***Government role**: None; the initiative was entirely internally funded and implemented.*<br>***Ownership**: Device/software IP remains with BioIntelliSense; Houston Methodist retains ownership of workflows, Epic integration, and the operational model.*

Alignment of Houston Methodist Continuous Vital Signs Monitoring (CVSM) Implementation Paper to JMIR's iCHECK-DH: Guidelines and Checklist for the Reporting on Digital Health Implementations

SECTION		ITEM	DESCRIPTION	TEXT EXTRACTED FROM MANUSCRIPT (WITH SUPPLEMENTARY CLARIFICATIONS IN *ITALICS*)<br>**Note**: All text in this column is drawn verbatim from the final clean manuscript. Supplementary clarifications are provided in *italics* solely for checklist readability; no new content has been introduced.
	13	Budget Planning (M)	Describe the planned budget for implementation (include costs such as change management, user training, project management, technology pricing, total cost of ownership). If possible, include actual costs, otherwise describe the range or percentage of the total budget. Indicate the period covered by the budget. Describe the budget for the intervention (e.g. development, purchase or adaptation of a free tool); if possible include real costs, otherwise describe as a percentage of the total budget. Indicate the duration covered by the budget.	"The program was funded internally as part of routine service redesign, with no direct patient costs. The budget included one-time implementation costs (devices, hubs, Epic integration, change management, user training, and project management) and ongoing operational costs (supply chain processes and device resupply). While exact expenditures are not reported, technology procurement and integration represented the largest share. The budget covered both the pilot year and enterprise rollout period, with upfront and ongoing investments establishing the foundation for a sustainable operating model."<br><br>***Funding source**: 100% internal Houston Methodist funding; no external or government funds.*<br>***Cost drivers**: Technology procurement and Epic integration were the largest budget categories.*<br>***Timeline**: Budget spanned pilot and enterprise rollout phases (2022–2024).*
	14	Sustainability (M)	Describe the Business model including the sustainability model (financial, economic, environment etc.). If possible, put outcomes in relation to cost to assess sustainability. Describe long term exit strategies, and all dimensions considered to sustain the project after the end of funding. If applicable, describe potential institutionalization of the project.	"Sustainability was achieved by embedding CVSM into standard operations, including VOC monitoring workflows, Epic documentation, supply chain logistics, and updated LMS training modules. Continued superuser engagement and institutional commitment of operational funding reinforced adoption. The internal business case projected nursing and PCA time savings as the key offset, supporting long-term sustainability and total cost of ownership. By fully institutionalizing CVSM into existing structures, Houston Methodist ensured long-term ownership and positioned the program as a core element of its broader digital care transformation strategy."<br><br>***Business model**: CVSM institutionalized as part of standard care operations, with internal funding covering ongoing costs.*<br>***Cost offsets**: Sustainability reinforced by projected nursing/PCA time savings that improve efficiency.*<br>***Exit strategy**: No exit strategy was required since the program was internally funded and fully absorbed into system operations.*
**RESULTS**	15	Coverage (M)	Describe whether the coverage of implementation is international, national, regional or at the level of e.g. municipalities. If coverage is sub-national, describe the regions. Provide information on the relative importance of the coverage (e.g. % of eligible population covered).	"Coverage ultimately included all adult, non-ICU inpatients across ~2,700 beds in the academic flagship, six community hospitals, and one long-term acute care facility, covering the full population of adult non-ICU inpatients system-wide."<br><br>***Geographic level**: Sub-national, regional coverage across the greater Houston area in Texas.*<br>***Population coverage**: Effectively 100% of eligible adult non-ICU inpatients across the health system.*
	16	Outcomes (M)	Primary and other outcome(s) of the implementation. Detail the actual outcomes, using the pre-defined outcome measures (if applicable).	"Primary outcomes were successful scale-up across all sites, reliable Epic integration, and sustained centralized monitoring. Device utilization exceeded 95%, with 100% staff training completion and ~50% of alerts resolved at the VOC review step, reducing frontline burden. Secondary outcomes included improved

Alignment of Houston Methodist Continuous Vital Signs Monitoring (CVSM) Implementation Paper to JMIR's [iCHECK-DH: Guidelines and Checklist for the Reporting on Digital Health Implementations]

SECTION	ITEM	DESCRIPTION	TEXT EXTRACTED FROM MANUSCRIPT (WITH SUPPLEMENTARY CLARIFICATIONS IN *ITALICS*)<br>**Note**: All text in this column is drawn verbatim from the final clean manuscript. Supplementary clarifications are provided in *italics* solely for checklist readability; no new content has been introduced.
			alert specificity, fewer patient refusals, increased staff acceptance, and operational efficiencies (nursing and PCA time savings).<br>Together, these outcomes demonstrated the feasibility and system-level value of CVSM at scale."<br><br>***Primary outcomes**: Enterprise-wide rollout (8 hospitals, ~2,700 beds), Epic integration, sustained VOC monitoring, >95% device utilization, 100% staff training completion, ~50% of alerts resolved at VOC review.*<br>***Secondary outcomes**: Reduced manual VS collection, ~4 nursing hours saved per shift, increased staff confidence, improved alert specificity, fewer patient refusals, greater staff acceptance.*<br>***Note**: No direct clinical outcomes (e.g., ICU transfers, mortality) were measured, as this was a service redesign quality improvement initiative.*
	17 Lessons learned (M))	Describe any lessons learned from the implementation experience that could be used to improve future outcomes. This could include, but is not limited to, success factors, implementation challenges or budget considerations.<br><br>Success factors: Describe factors that positively influenced the implementation (e.g. involvement of key stakeholders). Also describe contextual factors that may have positively influenced the results (e.g. new legal requirements that facilitated adoption).<br><br>Challenges to implementation: Describe challenges (process-related, such as resistance to change, but also technical). Include contextual factors that may have affected the achievement of outcomes such as an unexpected change of government, or 'opposing key players' who, despite potential participation, may hinder implementation (e.g. software companies managing regional digital health may act as barriers to innovation).	"Technical and workflow challenges identified during pilot informed enterprise rollout. Barcode scanning difficulties (poor label contrast) occasionally impeded device-to-patient matching; staff used packaging barcodes and WOW carts as interim workarounds, and the vendor improved barcode resolution. Rare 'double heart-rate' artifacts (concurrent S1/S2 detections) produced nonactionable alerts and were resolved via algorithm updates. Variation in device application sometimes caused skin irritation, addressed via re-education and adhesive remover spray. Early staff skepticism about using median CVSM values for documentation declined as experience accumulated and staff perceived CVSM values to be accurate and consistent with routine bedside measures; patient refusals similarly decreased with standardized education. These mitigations and training reinforcements were propagated system-wide during enterprise rollout."<br><br>***Success factors**: Early strategic alignment, phased rollout with iterative refinements, strong IT/monitoring infrastructure, sustained superuser/leadership engagement, and real-world clinical validation through illustrative case examples (Appendix 4), which demonstrated timely detection and escalation of patient deterioration.*<br>***Challenges**: Device/IT integration issues (barcode scanning, "double HR" artifacts), staff skepticism, patient hesitancy, and minor skin irritation.*<br>***Budget adherence**: Maintained; projected workforce efficiencies (nursing/PCA time savings) supported sustainability.*<br>***Recommendations**: Future implementations should adopt structured change management, close vendor collaboration, early piloting, and ongoing threshold optimization to maximize adoption and minimize alert fatigue.*

SECTION	ITEM		DESCRIPTION	TEXT EXTRACTED FROM MANUSCRIPT (WITH SUPPLEMENTARY CLARIFICATIONS IN *ITALICS*)<br>**Note**: All text in this column is drawn verbatim from the final clean manuscript. Supplementary clarifications are provided in *italics* solely for checklist readability; no new content has been introduced.
			Budget: Describe whether the implementation budget was adhered to, and if not, why not. Also detail the expected operational costs (e.g. licence, maintenance, human resources, updates to in-house developments) to estimate the total cost of ownership. Include real costs, otherwise describe them as a percentage of the total budget.<br><br>What recommendations can be drawn from the lessons learned?	
	18	Unintended consequences (NM[4])	Describe unintended consequences (positive or negative), harms or negative side-effects (if any).	"As noted above, unintended consequences including minor skin irritation, initial device hesitancy, and early false positives, were observed and through staff education, supplies, and vendor refinements; no significant harms were reported."<br><br>***Negative side effects**: Minor skin irritation from device adhesive, occasional false-positive alerts, and initial patient hesitancy.*<br>***Mitigation**: Addressed through re-education, adhesive remover supplies, and vendor algorithm updates.*<br>***Positive learning opportunity**: Early patient hesitancy prompted more proactive staff communication about the device's purpose and benefits, which not only reduced refusals but also strengthened patient engagement and trust.*
**DISCUSSION**	19	Conclusion (M)	Summary of the conclusions and future implications.	"This initiative demonstrates the feasibility and value of scaling CVSM across a large, diverse health system. Embedding continuous monitoring into standard workflows can improve patient safety, enhance staff efficiency, and build infrastructure for digitally enabled care models.<br>Future work should rigorously assess clinical outcomes, economic impact, and patient-reported measures, while exploring application in post-acute and ambulatory settings. As health systems address workforce shortages and rising acuity, wearable monitoring technologies like CVSM could serve as a core infrastructure for safer, more efficient, and patient-centered care."<br><br>***Summary**: CVSM was successfully institutionalized as a core operational model across eight hospitals.*<br>***Future implications**: Provides a scalable framework for other health systems; further evaluation is needed on clinical outcomes, cost-effectiveness, and patient-reported measures.*<br>***Broader alignment**: Supports health system digital transformation agendas by embedding continuous monitoring into enterprise workflows, offering lessons transferable to post-acute and ambulatory settings.*

[4] NM : Non-mandatory item

Alignment of Houston Methodist Continuous Vital Signs Monitoring (CVSM) Implementation Paper to JMIR's iCHECK-DH: Guidelines and Checklist for the Reporting on Digital Health Implementations

SECTION	ITEM		DESCRIPTION	TEXT EXTRACTED FROM MANUSCRIPT (WITH SUPPLEMENTARY CLARIFICATIONS IN *ITALICS*)<br>**Note**: All text in this column is drawn verbatim from the final clean manuscript. Supplementary clarifications are provided in *italics* solely for checklist readability; no new content has been introduced.
**GENERAL**	20	General (NM)	If applicable, include statement(s) on regulatory approvals (including, as appropriate, ethical approval, governance approval), trial or study registration (availability of protocol), and conflicts of interest. For implementation reports with a research component, ethical approval or a waiver from an appropriate ethics committee is required. For those without a research component, ethical considerations may still be relevant, but do not necessarily require approval or waiver. Authors may consult this article[5] for further guidance on ethical considerations in their specific context"	"The Institutional Review Board reviewed this initiative and deemed it 'Not Human Research,' as it represented a service redesign implemented as part of standard care.<br>This initiative was classified as a quality improvement initiative and 'Not Human Research' by the health system's Institutional Review Board. Data were extracted from the institution's internal database and are not publicly available due to privacy policies. No commercial funding was received, and the authors report no conflicts of interest."<br><br>***Regulatory/ethics****: IRB approval was not required; initiative classified as quality improvement and not human subjects research.*<br>***Governance****: Data governance and IT security approvals were completed internally (HIPAA-compliant).*<br>***Trial registration****: Not applicable; this was a service redesign initiative.*<br>***Conflicts of interest****: None reported by authors*.

[5] Eccles, M. P., Weijer, C., & Mittman, B. (2011). Requirements for ethics committee review for studies submitted to Implementation Science. Implementation science, 6(1), 1-3.
